# Tumor hyaluronan as a novel biomarker in non-small cell lung cancer: A retrospective study

**DOI:** 10.18632/oncotarget.28304

**Published:** 2022-11-02

**Authors:** Jun Gong, Michelle Guan, Haesoo Kim, Natalie Moshayedi, Sejal Mehta, Galen Cook-Wiens, Brent K. Larson, Jenny Zhou, Rishi Patel, Isaac Lapite, Veronica R. Placencio-Hickok, Richard Tuli, Ronald B. Natale, Andrew E. Hendifar

**Affiliations:** ^1^Gastrointestinal and Neuroendocrine Malignancies, Samuel Oschin Cancer Center, Cedars-Sinai Medical Center, Los Angeles, CA 90048, USA; ^2^Biostatistics and Bioinformatics Research Center, Cedars-Sinai Medical Center, Los Angeles, CA 90048, USA; ^3^Department of Pathology and Laboratory Medicine, Cedars-Sinai Medical Center, Los Angeles, CA 90048, USA; ^4^Department of Radiation Oncology, Samuel Oschin Cancer Center, Cedars-Sinai Medical Center, Los Angeles, CA 90048, USA; ^5^Department of Radiation Oncology, Memorial Sloan Kettering Cancer Center, New York, NY 10065, USA; ^6^Lung Cancer Research Program, Samuel Oschin Cancer Center, Cedars-Sinai Medical Center, Los Angeles, CA 90048, USA

**Keywords:** hyaluronan, hyaluronic acid, biomarker, prognostic, predictive

## Abstract

Introduction: Hyaluronan (HA) accumulation is associated with tumorigenesis and aggressive tumor behavior.

Aims: We investigated the biomarker potential of HA in non-small cell lung cancer (NSCLC).

Methods: HA levels were scored using affinity histochemistry in 137 NSCLC samples stratified by HA score ≤10, 11–20, 21–30, and >30 with HA-high defined as ≥25% expression in the extracellular matrix (ECM) of the tumor surface area. Overall survival (OS) and time to progression from initiation of taxane therapy (TTP) were compared using log-rank tests based on HA score.

Results: Of 122 patients with recurrent/metastatic NSCLC, 93 had mean HA scores that were not significantly different across clinicopathologic variables. Frequency of HA-high tumors did not differ by histology (34/68 adenocarcinomas vs. 12/25 squamous tumors, Fisher’s *p* = 1.0000). Median OS for recurrent/metastatic adenocarcinoma was 35.5 months (95%, 23.6–50.3) vs. 17.9 months for squamous (95%, 12.7–37.0, log-rank test, *p* = 0.0165). OS was not significantly different by HA quartiles, high or low (<25) HA score and tumor histology, and HA biopsy site (all *p* > 0.05). Median TTP (*n* = 98) significantly differed by HA quartile (2.8 months for HA score ≤10; 5.0 months for 11–20; 7.9 months for 21–30; 3.9 months for >30, *p* = 0.0265). Improved TTP trended in HA-high over HA-low tumors (*n* = 98, *p* = 0.0911).

Conclusion: In this NSCLC cohort, tumor HA level represents a potential biomarker for TTP, which remains a cornerstone of NSCLC therapy. Further validation is warranted to identify the HA accumulation threshold associated with clinical benefit.

## INTRODUCTION

In 2012, an estimated 1.8 million new cases of lung cancer occurred resulting in an estimated 1.5 million deaths in both men and women worldwide [1]. Globally, lung cancer remains the leading cause of cancer-related death in men and the second-leading cause of cancer death in women, underscoring the need to further develop preventive, diagnostic, prognostic, and therapeutic strategies to improve outcomes [1].

Hyaluronic acid or hyaluronan (HA, molecular weight ranging from 10^5^–10^7^ Dalton) is an uncomplicated, free unbranched glycosaminoglycan (GAG comprised of 2,000–25,000 repeating disaccharide units of N-acetyl-glucosamine and D-glucuronic acid and is primarily synthesized by integral plasma membrane proteins known as hyaluronan synthases (HAS1-3) [2]. HA constitutes an important component of the extracellular matrix (ECM) in vertebrate tissues with unique physiochemical properties that enable pliable tissue remodeling and cell motility [2]. Furthermore, binding of HA to cell surface receptors including CD44, receptor for hyaluronan-mediated motility (RHAMM), and Toll-like receptor-4 facilitates intracellular signaling transduction involved in cell proliferation, aggregation, angiogenesis, and migration [2].

Recently, HA has been shown to contribute to the tumorigenesis of a number of malignancies [2]. Notably, the magnitude of HA accumulation around tumor cells and surrounding stroma has been shown to strongly correlate with cancer aggressiveness through enhancement of tumor cell proliferation, invasion, angiogenesis, metastasis, and tumor-stroma interactions [3]. HA production has also demonstrated the ability to induce epithelial to mesenchymal transition (EMT) and therapeutic resistance in preclinical non-small cell lung cancer (NSCLC) models [4].

The purpose of this study was to evaluate the significance of HA as a potential biomarker in NSCLC. Specifically, we investigated the prognostic and predictive value of tumor HA levels in a large cohort of predominantly advanced-stage NSCLC patients.

## RESULTS

### Study population

174 NSCLC patients were screened for this study (Table 1). The majority of patients had adenocarcinomas (64.9%), stage IV (44.8%), or recurrent (25.3%) disease. Tumor HA staining was performed on 137 cases (78.7%). Of the 122 patients with recurrent or metastatic NSCLC, 93 had HA scores (68 on tumor samples at baseline or pretreatment) with 53 biopsies performed on primary tumors (43.4%).

**Table 1:** Patient characteristics

Characteristic (*n* = 174)	Frequency (%)
Gender	
Female	82 (47.1%)
Male	92 (52.9%)
Histology	
Adenocarcinoma	113 (64.9%)
Squamous	55 (31.6%)
Adenosquamous	6 (3.5%)
Tumor HA score available	137 (78.7%)
Adenocarcinoma with HA scores ≥25	42/90 (46.7%)
Squamous/adenosquamous with HA scores ≥25	25/47 (53.2%)
All histologies with HA scores <25	70 (51.1%)
Stage IV (AJCC 7th Edition)	78 (44.8%)
Recurrent disease	44 (25.3%)
**Recurrent or metastatic disease (*n* = 122)**	**Frequency (%)**
Race	
Caucasian	85 (69.7%)
Middle Eastern	12 (9.8%)
Asian	12 (9.8%)
African-American	10 (8.2%)
Hispanic	3 (2.5%)
ECOG performance status	
0–1	95 (77.9%)
≥2	11 (9.0%)
NR	16 (13.1%)
Biopsy site	
Primary	53 (43.4%)
Metastatic	43 (35.3%)
NR	26 (21.3%)
Line(s)(*n* of therapy at biopsy)	
0	68 (55.7%)
1–2	22 (18.0%)
3–4	2 (1.7%)
NR	30 (24.6%)
Mutation status	
*KRAS*	17 (13.9%)
*EGFR*	13 (10.7%)
*ALK*	1 (0.82%)
Pathologic grade	
1–2	32 (26.2)
3–4	83 (68.0%)
NR	7 (5.8%)
Tumor HA score available	93 (76.2%)
Adenocarcinomas with HA scores ≥25	34/68 (50.0%)
Squamous/adenosquamous with HA scores ≥25	12/25 (48.0%)^a^
All histologies with HA scores <25	47 (50.5%)

Abbreviations: HA: hyaluronic acid; AJCC: American Joint Committee on Cancer; ECOG: Eastern Cooperative Oncology Group; NR: not reported or unavailable. ^a^Fisher’s exact test (two-tailed) did not show any significant difference between frequency of squamous/adenosquamous tumors with high HA scores compared to frequency of adenocarcinoma tumors with high HA scores (*p* = 1.0000).

### Tumor HA scores by clinicopathologic variables

Among all NSCLC patients, there were no significant differences between various clinicopathologic factors and having a high (≥25) or low (<25, Table 2) HA score. Specifically, there were no significant associations between low or high tumor HA scores according to pathologic grade (Fisher’s exact test, two-tailed *p* = 0.7105), gender (*p* = 1.0000), race (*p* = 0.7984), *KRAS* mutation status (*p* = 1.0000), *EGFR* mutation status (*p* = 0.7524), family history of cancer (*p* = 0.3989), Eastern Cooperative Oncology Group (ECOG) performance status (PS, *p* = 0.3288), HA biopsy site (*p* = 0.3952), and line of therapy at the time of biopsy (*p* = 0.6619). Furthermore, there were no significant differences in mean and median HA scores based on pathologic grade (Kruskal-Wallis test, *p* = 0.9559), race (*p* = 0.7143), *KRAS* status (*p* = 0.6019), *EGFR* status (*p* = 0.3349), family history of cancer (*p* = 0.3881), ECOG PS (*p* = 0.4955), HA biopsy site (*p* = 0.514), and line of therapy at time of biopsy (*p* = 0.3229, Table 3).

**Table 2:** Comparison of clinicopathologic variables by low and high tumor HA scores

Characteristic	High HA score (≥25)	Low HA score (<25)	Fisher’s Exact test (two-tailed *p*-value)
Pathologic grade	*n* = 43	*n* = 45	0.7105
1	2 (4.65%)	1 (2.22%)	
2	11 (25.58%)	16 (35.56%)	
3	19 (44.19%)	19 (42.22%)	
4	11 (25.58%)	9 (20%)	
Gender	*n* = 46	*n* = 47	1.0000
Female	24 (52.17%)	24 (51.06%)	
Race	*n* = 46	*n* = 47	0.7984
Caucasian	31 (67.39%)	30 (63.83%)	
African-American	5 (10.87%)	4 (8.51%)	
Asian	6 (13.04%)	5 (10.64%)	
Hispanic	1 (2.17%)	1 (2.13%)	
Middle Eastern	3 (6.52%)	7 (14.89%)	
Mutation status			
*KRAS* WT+MT	*n* = 24	*n* = 24	
*KRAS*	6 (25%)	5 (20.83%)	1.0000
*EGFR* WT+MT	*n* = 35	*n* = 35	
*EGFR*	7 (20%)	5 (14.29%)	0.7524
Family history^a^	*n* = 45	*n* = 46	0.3989
Positive	24 (53.3%)	29 (63.04%)	
ECOG PS	*n* = 38	*n* = 41	0.3288
0	10 (26.32%)	16 (39.02%)	
1	21 (55.26%)	23 (56.1%)	
2	4 (10.53%)	1 (2.44%)	
3	2 (5.26%)	1 (2.44%)	
4	1 (2.63%)	0 (0%)	
HA biopsy site	*n* = 43	*n* = 46	0.3952
Metastatic	17 (39.53%)	23 (50%)	
Primary	26 (60.47%)	23 (50%)	
Line(s) of therapy at biopsy	*n* = 43	*n* = 44	0.6619
1	7 (16.28%)	9 (20.45%)	
2	3 (6.98%)	1 (2.27%)	
3	0 (0%)	1 (2.27%)	
4	1 (2.33%)	0 (0%)	
None	32 (74.42%)	33 (75%)	

Abbreviations: HA: hyaluronic acid; WT: wild-type; MT: mutant; ECOG PS: Eastern Cooperative Oncology Group performance status. ^a^Any cancer.

**Table 3:** Mean ranks of tumor HA scores by clinicopathologic variables

Characteristic	Mean HA score ± SD	Median HA score	Kruskal Wallis test *p*-value
Pathologic grade (*n* = 88)			0.9559
1 (*n* = 3)	30 ± 20	30	
2 (*n* = 27)	26.11 ± 16.13	20	
3 (*n* = 38)	24.87 ± 15.13	22.5	
4, *n* = 20	26.25 ± 18.34	25	
Race (*n* = 93)			0.7143
Caucasian (*n* = 61)	25.66 ± 15.23	25	
African-American (*n* = 9)	30.56 ± 23.24	25	
Asian (*n* = 11)	26.82 ± 17.36	25	
Hispanic (*n* = 2)	22.5 ± 3.54	22.5	
Middle Eastern (*n* = 10)	19.5 ± 15.36	15	
Mutation status (*n* = 172)			
KRAS MT (*n* = 11)	22.27 ± 13.11	25	0.6019
KRAS WT (*n* = 37)	27.03 ± 17.58	20	
EGFR MT (*n* = 12)	30.83 ± 19.29	30	0.3349
EGFR WT (*n* = 58)	25.34 ± 15.72	20	
ALK (all ALK WT, *n* = 54)	27.41 ± 16.53	25	–
Family history (*n* = 53)^a^	24.72 ± 17.28	20	0.3881
No family history (*n* = 38)	26.18 ± 14.54	25	
ECOG PS (*n* = 79)			0.4955
0 (*n* = 26)	20.96 ± 12.25	20	
1 (*n* = 44)	25.11 ± 17.37	20	
2 (*n* = 5)	30 ± 15.41	25	
3 (*n* = 3)	33.33 ± 20.82	40	
4 (*n* = 1)	30 ± –	30	
HA biopsy site (*n* = 89)			0.5104
Metastatic (*n* = 40)	24.5 ± 15.43	20	
Primary (*n* = 49)	26.63 ± 17.3	25	
Line(s) of therapy at biopsy (*n* = 87)			0.3229
1 (*n* = 16)	22.19 ± 15.6	20	
2 (*n* = 4)	42.5 ± 20.62	40	
3 (*n* = 1)	20 ± –	20	
4 (*n* = 1)	40 ± –	40	
None (*n* = 65)	25.69 ± 16.3	20	

Abbreviations: HA: hyaluronic acid; WT: wild-type; MT: mutant; ECOG PS: Eastern Cooperative Oncology Group performance status. ^a^Any cancer.

### Overall survival analyses in recurrent or metastatic disease cohort

**Figure 1:**
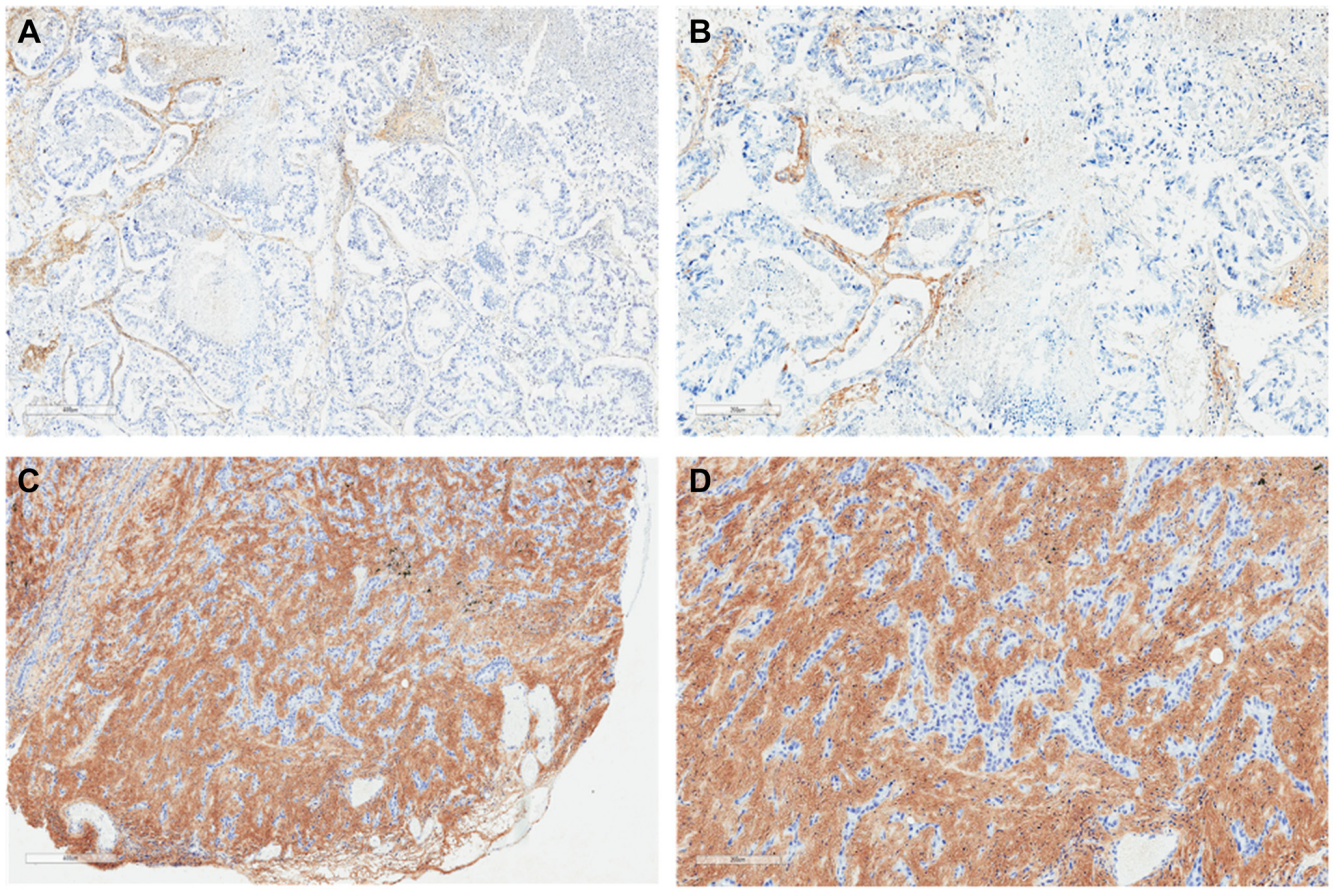
Lung adenocarcinoma histochemistry. Representative sections illustrating tumor HA histochemistry staining (Ventana HA RxDx Assay) in FFPE lung adenocarcinoma samples scored as HA-low at 6X (**A**) and 11.2X (**B**) magnifications and HA-high at 6X (**C**) and 11.2X (**D**) magnifications. HA expression in the ECM of ≥25% of the tumor surface area at any intensity was designated as HA-high.

**Figure 2:**
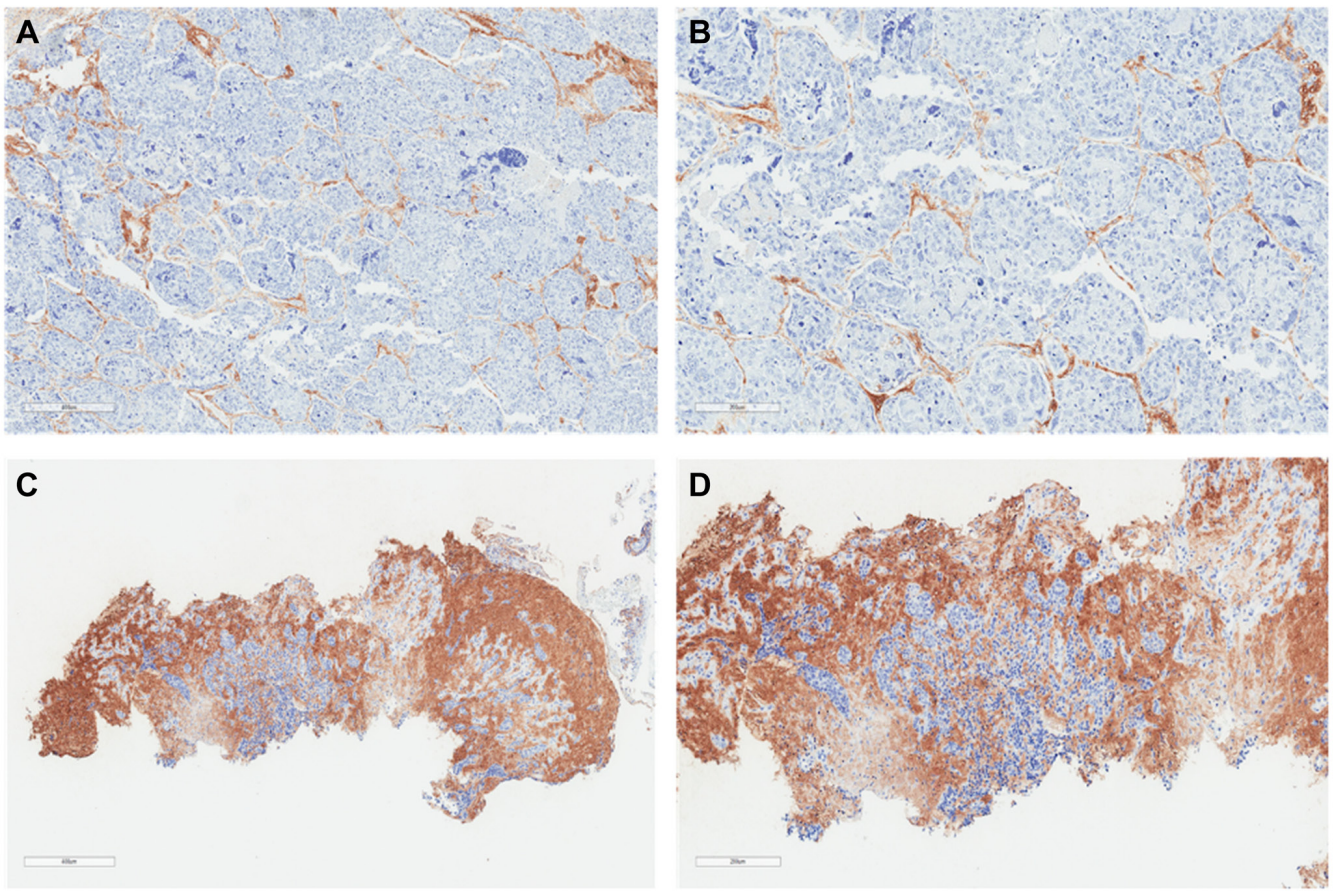
Lung squamous cell carcinoma histochemistry. Representative sections illustrating tumor HA histochemistry staining (Ventana HA RxDx Assay) in FFPE lung squamous cell carcinoma samples scored as HA-low at 6X (**A**) and 11.2X (**B**) magnifications and HA-high at 6X (**C**) and 11.2X (**D**) magnifications. HA expression in the ECM of ≥25% of the tumor surface area at any intensity was designated as HA-high.

**Figure 3:**
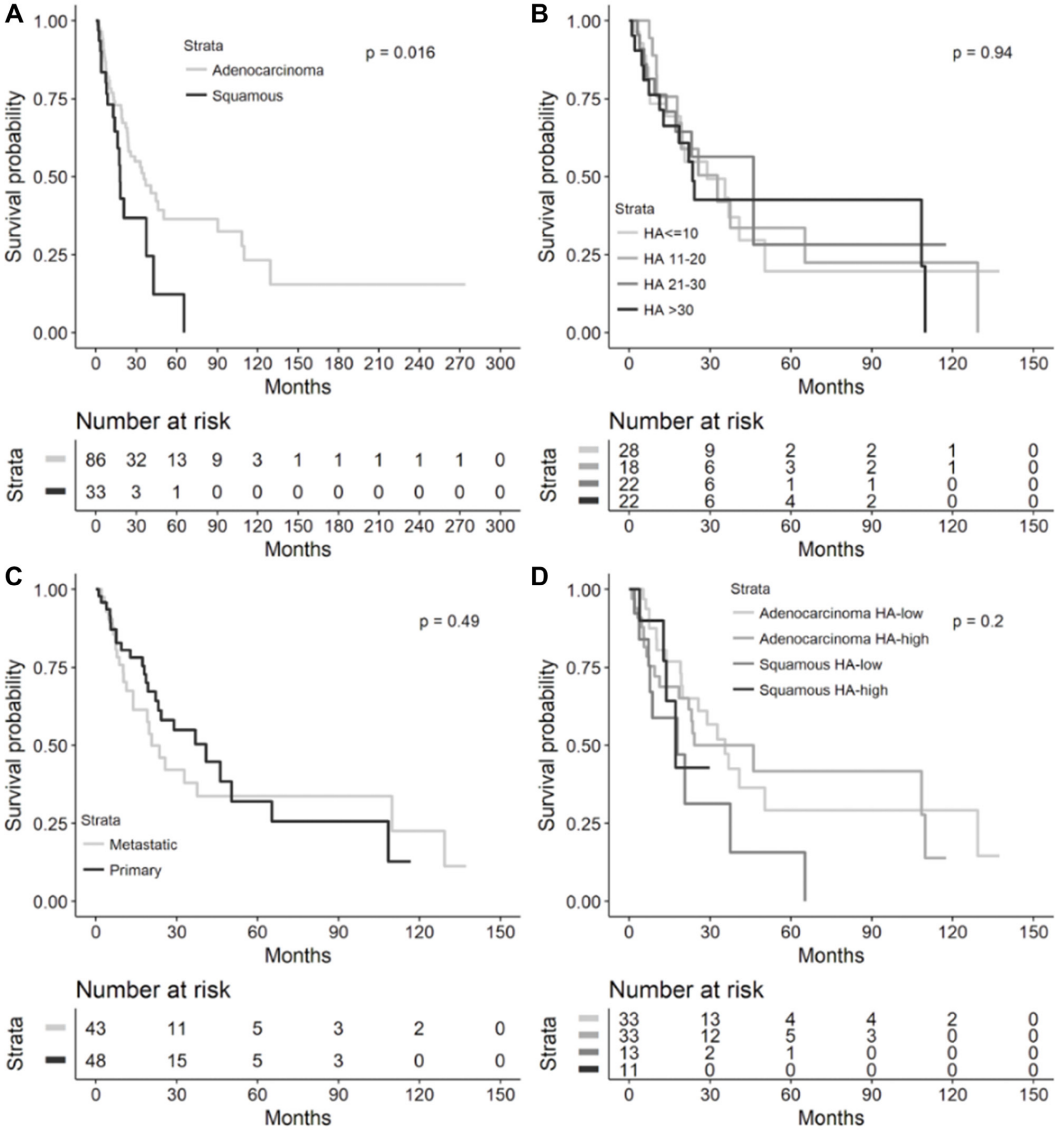
Overall survival in metastatic or recurrent NSCLC cohort. Kaplan-Meier overall survival curves from diagnosis to death in the metastatic or recurrent NSCLC cohort by (**A**) lung cancer histology (*n* = 119), (**B**) HA score quartiles (*n* = 90), (**C**) HA biopsy site (*n* = 91), and (**D**) histology and HA-high (≥25) or HA-low (<25, *n* = 90). Median overall survival provided in each figure inset. Abbreviations: NSCLC: non-small cell lung cancer; HA: hyaluronic acid; CI: confidence interval.

In the cohort of patients with recurrent or metastatic NSCLC, the median OS for adenocarcinoma was 35.5 months (95% confidence interval (CI) 23.6–50.3, Figure 1) compared to 17.9 months for squamous cell carcinoma (SCC) histology (95% CI 12.7–37.0, Figure 2, log-rank test, *p* = 0.0165). As seen in Figure 3, OS was not significantly different by HA score quartiles (HA score ≤10 median OS 28.9 (95% CI 13.9–50.3), HA score 11–20 median OS 32.8 (95% CI 10.3–65.2), HA score 21–30 median OS 46.2 (95% CI 13.8-infinity), and HA score >30 median OS 23.6 (95% CI 11.4–109.9), log-rank *p* = 0.9422)), OS also did not differ by HA biopsy site (metastatic median OS 20.7 (95% CI 13.8–109.9) vs. primary median OS 40.9 (95% CI 22.1–65.2), log-rank *p* = 0.4928)), or by high (≥25) vs. low (<25) HA score and tumor histology (adenocarcinoma/HA-low median OS 35.5 (95% CI 19.3–50.3), adenocarcinoma/HA-high median OS 24.3 (95% CI 11.4–109.9), squamous/HA-low median OS 17.9 (95% CI 3.8–37.5), and squamous/HA-high median OS 17.2 (95% CI 4-infinity), log-rank *p* = 0.1953)).

### Overall survival analyses in all patients

**Figure 4:**
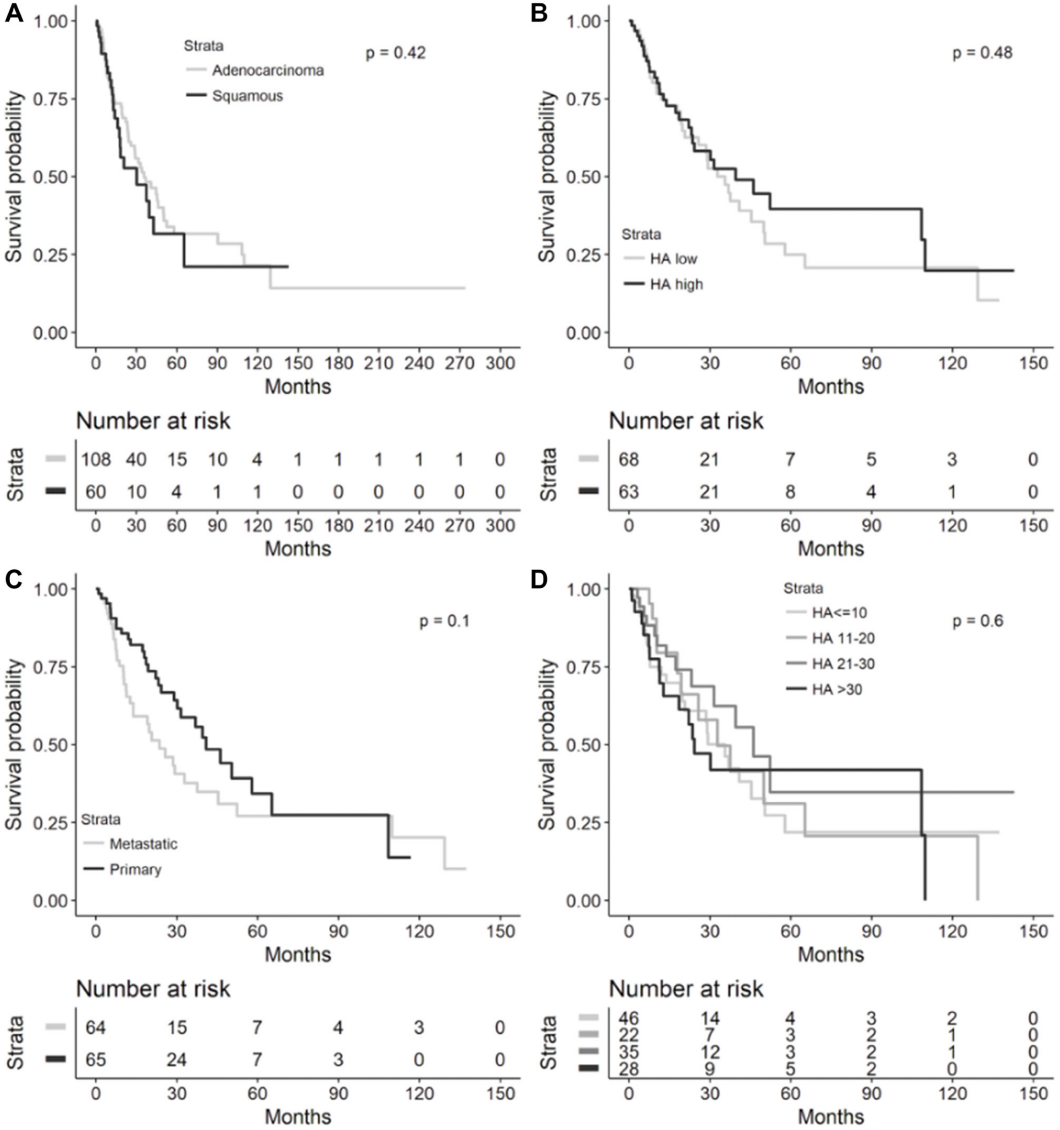
Overall survival in overall NSCLC cohort. Kaplan-Meier overall survival curves from diagnosis to death in the overall NSCLC cohort by (**A**) lung cancer histology (*n* = 168), (**B**) High (≥25) or low (<25) HA score (*n* = 131), (**C**) HA biopsy site (*n* = 129), and (**D**) HA score quartiles (*n* = 131). Median overall survival provided in each figure inset. Abbreviations: NSCLC: non-small cell lung cancer; HA: hyaluronic acid; CI: confidence interval.

In the overall cohort, there were no significant differences in OS based on tumor histology, low or high HA score, HA biopsy site, and HA score quartiles (Figure 4). Median OS was 35.5 months (95% CI 25.7–49.9) for NSCLC patients with adenocarcinoma vs. 30.2 months (95% CI 15.9–65.2, log-rank *p* = 0.4244) with SCC histology, 32.8 months (95% CI 20.7–49.9) for HA-low tumors vs. 39.6 months (95% CI 23.2–109.9, log-rank *p* = 0.4824) for HA-high tumors, 23.6 months (95% CI 12.6–37.5) for metastatic HA biopsy site vs. 40.9 months (95% CI 28.9–65.2, log-rank p 0.1026) for primary HA biopsy site, and 35.5 months (95% CI 19.7–50.3) for HA score ≤10, 32.8 months (95% CI 17.9–65.2) for HA score 11–20, 46.2 months (95% CI 23.2-infinity) for HA score 21–30, and 24.3 months (95% CI 11.4–109.9, log-rank *p* = 0.5994) for HA score >30.

### Time to progression (TTP) analyses

**Figure 5:**
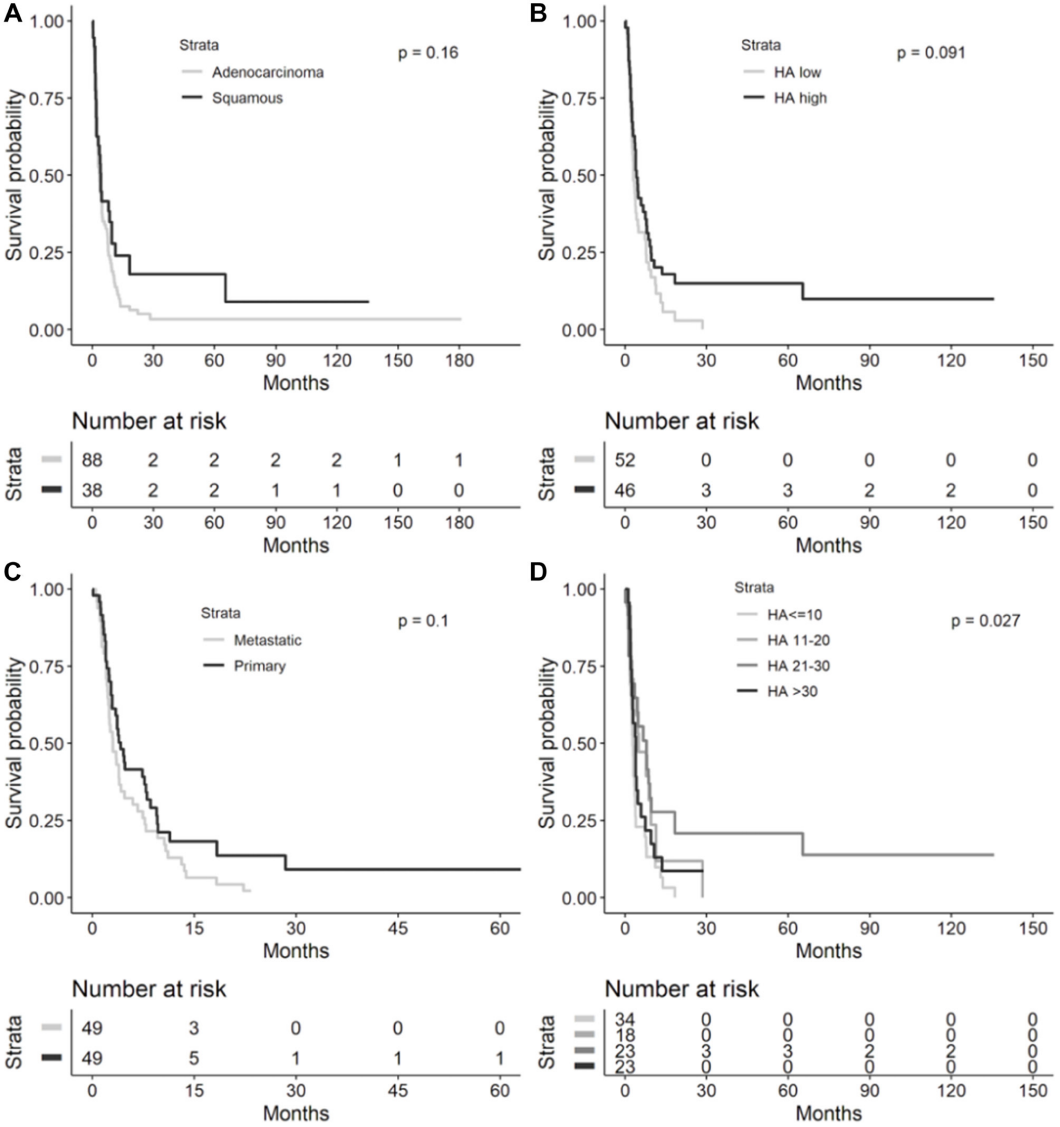
Time to progression in overall NSCLC cohort. Kaplan-Meier curves from initiation of taxane therapy to progression (taxane TTP) in the overall NSCLC cohort by (**A**) lung cancer histology (*n* = 126), (**B**) HA biopsy site (*n* = 98), (**C**) High (≥25) or low (<25) HA score (*n* = 98), and (**D**) HA score quartiles (*n* = 98). Median taxane TTP provided in each figure inset. Abbreviations: NSCLC: non-small cell lung cancer; HA: hyaluronic acid; CI: confidence interval.

A total of 126 NSCLC patients in the cohort were treated with taxanes at any time during their management and were included for TTP analyses (Figure 5). Taxane TTP did not significantly differ based on tumor histology (adenocarcinoma median TTP 3.7 (95% CI 2.8–4.7) vs. squamous median TTP 4.2 (95% CI 2.1–9.6, log-rank *p* = 0.1641)) and HA biopsy site (metastatic median TTP 3 (95% CI 2.3–4.2) vs. primary median TTP 4.2 (95% CI 3–7.9, log-rank *p* = 0.1020)). Median taxane TTP was not significantly higher in those with HA-high tumors (median TTP 4.2 (95% CI 2.8–7.5)) compared to HA-low tumors (median TTP 3.1 (95% CI 2.5–4.2, log-rank *p* = 0.0911)). Median taxane TTP was significantly different by HA quartiles (2.8 months, 95% CI 1.9–3.5 for HA score ≤10, 5.0 months, 95% CI 2.2–9.6 for 11–20, 7.9 months, 95% CI 2.5–9.7 for 21–30, and 3.9 months, 95% CI 2.4–4.7 for >30, log-rank *p* = 0.0265).

## DISCUSSION

In this study, we retrospectively assessed the relationship between tumor HA levels and various clinicopathologic factors as well as survival in a cohort of predominantly advanced-stage NSCLC patients. To the best of our knowledge, there have only been 2 other studies (both retrospective) investigating the prognostic potential of HA in NSCLC patients [5, 6]. In one study from Finland, HA staining was performed on tumor specimens from 226 cases of NSCLC and identified no significant differences in low or high HA staining in the cancer cells or tumor stroma across clinicopathologic factors including histologic grade, stage, and tumor size [5]. Notably, adenocarcinomas in the majority of cases showed a low percentage of HA-positive cancer cell staining (79% of cases) compared to SCCs (8% of cases, *p* < 0.0001) and a low percentage of cancer cell-positive HA expression was significantly associated with higher probability of disease recurrence (*p* = 0.0300); neither of these findings reached statistical significance, however, when HA staining intensity was excluded to the tumor stroma only [5].

A Brazilian retrospective study involving HA staining on tumor specimens from 46 NSCLC cases similarly identified a significant association between strong HA staining in cancer cells and SCCs (32.1%) compared to adenocarcinomas (17.9%, *p* < 0.0010) though again there was no significant difference in strong HA staining between SCCs and adenocarcinomas (*p* = 0.3000) when analysis was limited to the peritumoral stroma [6]. Our findings are similar to the Finland report in that we did not observe any significant differences in low/high HA scores or mean/median HA scores across various clinicopathologic factors (Tables 2 and 3). We observed that 42/90 (46.7%) adenocarcinomas had high HA scores (≥25) compared to 25/47 (53.2%) of SCC/adenosquamous tumors with high HA scores in our overall cohort, and 34/68 (50.0%) of adenocarcinomas were HA-high compared to 12/25 (48.0%, Fisher’s exact two-tailed *p* = 1.0000) of SCC/adenosquamous tumors that were HA-high in our recurrent and metastatic NSCLC cohort (Table 1). Our findings are in contrast to the HA staining findings in cancer cells of both the Finland and Brazilian studies but are consistent with the HA staining patterns in the peritumoral stroma in both studies [5, 6].

It is worthwhile to mention that we observed a significantly improved OS in patients with adenocarcinoma compared to SCC in the recurrent and metastatic NSCLC cohort, but no significant difference in OS between these 2 histologies in our overall NSCLC cohort (Figures 3 and 4). This is consistent with findings from large population-based registries that have shown significantly higher survival in patients with stage IV adenocarcinomas of the lung compared to stage IV SCCs of the lung [7]. However, in cohorts with resected or non-metastatic NSCLC, differences in outcomes based on adenocarcinoma or SCC histology have been mixed, potentially owing to impact on survival that is stage-dependent, patient characteristic-dependent, and tumor biology-dependent that has yet to be further resolved in stage I-III NSCLC – these factors certainly could have affected the lack of a significant OS difference across histologies in our overall cohort [8–12].

Furthermore, in the Finland study, a low percentage of HA-positive cancer cells predicted a shortened disease-free survival (DFS, *p* = 0.05) overall in univariate analysis, but adenocarcinomas trended towards poor OS (*p* = 0.07) in tumors with a high percentage of cancer cell-associated HA [5]. Cancer cell-associated HA staining had no prognostic value for DFS (*p* = 0.02) in multivariate analysis of all cases, but in adenocarcinomas, a strong HA signal in the tumor stroma significantly predicted shorter DFS in both univariate and multivariate analyses [5]. In the Brazilian study, a high percentage of HA expression in cancer cells significantly predicted shortened DFS (*p* = 0.02), while adenocarcinomas with a high percentage of cancer cell-associated HA staining exhibited a trend toward diminished OS (*p* = 0.09) on univariate analysis [6]. Interestingly, on multivariate analysis controlled for adenocarcinoma histology, a low cancer cell-associated HA signal significantly correlated with better survival (hazard ratio (HR) 0.08, *p* = 0.0300) [6]. In our overall and recurrent/metastatic NSCLC cohorts, the quartile with the highest HA levels (>30) had the shortest median OS compared to other HA staining quartiles but did not reach statistical significance (Figures 3 and 4). A comparison of OS between low-HA and high-HA tumors similarly did not reach statistical significance in the overall cohort (Figure 4). In the recurrent/metastatic cohort, HA-high tumors (≥25) had shorter median OS compared to HA-low tumors within the same histologic classification, but squamous histology tumors with both HA-high and HA-low levels had shorter median OS compared to adenocarcinomas with either HA-high or HA-low levels (Figure 3); it is likely that the difference in OS seen in advanced-stage adenocarcinomas compared to SCCs, in general, contributed to the OS difference seen across advanced-stage NSCLC histologies regardless of HA staining intensity.

There are several plausible explanations for the different survival findings seen in our study compared to the other 2 studies on the prognostic value of HA staining in NSCLC. The Finland and Brazilian studies utilized a biotinylated HA probe in FFPE tissue samples [5, 6], while in our study we evaluated tumor HA levels using a novel recombinant HA binding probe altered to incorporate a rabbit fragment crystallizable (Fc) region with high specificity and sensitivity for binding HA in tissues (co-developed by Ventana Medical Systems, Inc. and Halozyme Therapeutics) [13]. Additionally, the former studies used different thresholds for declaring HA-high and HA-low tumors. In the Finland study, HA staining was considered high if ≥30% of the tumoral area showed persistent HA signal, while <30% HA staining was considered low for analysis in cancer cells [5]. HA staining in the peritumoral stroma, however, was classified as low with <50% of tissue having intense signal or high with ≥50% signal [5]. In the Brazilian study, having <48% of the area in cancer cells and <84% in the peritumoral stroma with persistent HA signal were considered HA-low, while having ≥48% and ≥84%, respectively, were considered HA-high [6]. In our cohort, we focused HA staining in the ECM and designated having ≥25% of the tumor surface area at any intensity as HA-high and HA score <25% as HA-low. Furthermore, differences in patient demographics and disease characteristics across studies could have accounted for variability in findings. For example, our study investigated a predominantly advanced-stage, U.S.-based NSCLC population while the majority of the Finland cohort was stage I (64.4%) and 100% of the Brazilian cohort was non-metastatic [5, 6].

Indeed, a growing body of evidence supports that stronger HA staining intensity is prognostic of worse survival across several types of tumors though with exceptions; the prognostic value of HA staining also appears to be dependent on tissue type in which HA staining is performed (e.g., effusion or tumor tissue), location of HA staining (e.g., tumor stroma or cancer cell), and HA staining pattern (e.g., homogenous or irregular) [14–18]. In addition to the above variables, the retrospective design of our study, heterogeneity in patient characteristics and tumor biology, and relatively small number of events in some of our survival analyses may have contributed to the lack of statistically significant OS differences seen across HA scores. Nevertheless, our findings can inform future investigations of the prognostic value of tumor HA levels in NSCLC. Specifically, future studies of ideally large, prospective design seeking to validate these findings should take into account factors including tumor histology, disease stage, HA staining threshold, pattern, and location, HA probe, and other disease characteristics that can lead to variability in predicting outcomes. Furthermore, levels of HA signaling pathway mediators and regulators of HA synthesis such as RHAMM, CD44, hyaluronidases, and hyaluronan synthases have shown prognostic potential in lung cancer warranting further validation [19–22]. Ideally, incorporation of tumor HA levels along with assessment of upstream and downstream regulators of HA can produce a comprehensive HA signature or panel that demonstrates greater biomarker potential.

Lastly, given the heterogeneity of treatments documented in our cohort, we performed additional survival analyses, namely TTP analyses, in patients treated with taxanes that remains a cornerstone in the systemic therapy of NSCLC in localized and metastatic settings. We observed that taxane TTP was significantly different across HA quartiles (Figure 5) with the shortest median taxane TTP of 2.8 months seen in tumors with the lowest HA scores (≤10), while longer median taxane TTP was seen in those with HA scores of 11–20 (5.0 months) and 21–30 (7.9 months). Of note, the lower TTP seen in tumors with HA scores >30 (3.9 months) may have been limited by virtue of having the fewest number of risk events in this group. Additionally, we recognized that taxane TTP improved in HA-high tumors (≥25) compared to that in HA-low tumors (<25, *p* = 0.0911).

The reason for improved TTP to taxanes with higher tumor HA scores in our cohort is unclear, but HA has been shown to sensitize cancer cells and tumors in animal models to chemotherapy [23]. Our findings are hypothesis-generating and warrant further validation, particularly as HA assessment may serve to predict therapeutic benefit in high-HA lung tumors, a patient subset who may have an adverse prognosis. Indeed, preclinical evidence supports that HA can potentially reverse resistance to paclitaxel and combination HA and taxane-based therapy enhances antitumor efficacy in lung cancer models [24–26]. Prospective clinical evidence demonstrated significant improvements in progression-free survival (PFS) in untreated metastatic pancreatic tumors that are HA-high (≥50%) treated with the combination of pegvorhyaluronidase alfa (PEGPH20, HA-targeting agent) and nab-paclitaxel/gemcitabine over the same chemotherapy alone. This phase II trial demonstrated that tumor HA may serve as a clinically-relevant predictive biomarker.

Growing evidence also supports that CD44-HA and/or RHAMM-HA signaling putatively promotes tumorigenesis by activating growth factor receptor (e.g., ErbB2 and c-Met), PI3K/Akt and Erk, small GTPase protein (e.g., RhoA and Rac1), Ras, and NFkB and Src signaling, while p38 mitogen-activated protein kinase-HA-dependant reprogramming of the TME drives lung tumorigenesis – these findings open potential avenues to combine targeted agents against these molecular targets with HA-targeted therapies and/or other systemic therapies [27, 28].

### Key messages

- Tumor HA level represents a potential biomarker for taxane therapy, which remains a cornerstone of systemic therapy in NSCLC.
- Median OS for recurrent/metastatic adenocarcinoma was 35.5 months vs. 17.9 months for squamous.
- Median taxane TTP significantly differed by HA quartile.

## MATERIALS AND METHODS

### Study population

Patients with NSCLC treated at the Samuel Oschin Cancer Center at Cedars-Sinai Medical Center (Los Angeles, CA, USA) between October 2006 and September 2016 were screened for eligibility: those with histologic diagnoses of NSCLC and archival tissue specimens available for tumor HA staining. There were no exclusions based on disease stage, pathologic grade, site of biopsy, *KRAS*, *EGFR*, or *ALK* mutation status, medical comorbidities, performance status, previous treatments (surgical, radiation, or systemic therapy), or lines of prior therapy. The study was approved by the Institutional Review Board (IRB) Pro00039754.

### Tissue samples and tumor hyaluronan staining

Tumor HA staining was performed on archival formalin-fixed, paraffin-embedded (FFPE) tissue according to institutional guidelines. Tumor HA levels were assessed and scored using affinity histochemistry (Ventana HA RxDx Assay; Roche, Tucson, AZ) in the tumor microenvironment (TME) as previously described [29]. In brief, the tumor extracellular matrix (ECM) that stained for HA above background was recorded as a proportion of the total tumor surface area (Figures 1 and 2). HA expression in the ECM of ≥25% of the tumor surface area at any intensity was designated as HA-high given that at this cutoff roughly 50% of patients with available HA data would have low or high HA scores. HA scores were stratified into quartiles (HA score ≤10, 11–20, 21–30, and >30) based on roughly equivalent distribution of HA scores in patients with available HA data for additional analyses. Tumor HA scores obtained from biopsies at diagnosis or earliest time point along treatment continuum, if biopsy samples at initial diagnosis were unavailable, were utilized.

### Statistical analyses

Descriptive statistics were used to characterize patient characteristics abstracted by medical record review and expressed as frequencies and percentages. Continuous data were summarized with mean, standard deviation, and median values. Overall survival (OS) and time to progression from initiation of taxane therapy (taxane TTP) were compared using log-rank tests based on HA score and clinicopathologic factor. Mean ranks of HA scores were analyzed per patient characteristic using the Kruskal-Wallis test. Fisher’s exact test (two-tailed *p*-value) was used to compare differences in clinicopathologic variables across low and high tumor HA scores. A *p*-value < 0.05 was considered statistically significant.
